# Perceptual and Acoustical Features of Dysarthria in Essential Tremor: An Observational Study that Expands the Cerebellar Features of Essential Tremor

**DOI:** 10.5334/tohm.1180

**Published:** 2026-05-06

**Authors:** Nayanika Ghosh, Nora Hernandez, Ethan Wainman, Isabella Forestieri, Elan D. Louis, Rosemary A. Lester-Smith

**Affiliations:** 1 Department of Speech, Language, and Hearing Sciences, Moody College of Communication, The University of Texas at Austin, Austin, TX, USA; 2 Department of Neurology, University of Texas Southwestern Medical Center, Dallas, Texas, USA; 3 Peter O’Donnell Jr. Brain Institute, University of Texas Southwestern Medical Center, Dallas, Texas, USA

**Keywords:** essential tremor, dysarthria, cerebellar features

## Abstract

**Background:**

Clinical, neuroimaging and postmortem studies indicate cerebellar dysfunction in essential tremor (ET). Clinically this is reflected in intention tremor, gait ataxia, and impaired motor timing. However, the effect of cerebellar dysfunction on speech in ET remains unclear, and research on dysarthria in ET is limited. This study aimed to 1) identify the perceptual features of dysarthria in ET, 2) verify the prominent perceptual features using acoustical analyses, and 3) determine the percentage of participants exhibiting hyperkinetic, ataxic or mixed hyperkinetic-ataxic dysarthria.

**Methods:**

Speech samples from fifteen participants with ET were analyzed. An expert rater completed auditory-perceptual ratings, and acoustical analyses were completed that corresponded to perceptual features detected in participants’ speech, including speech timing, prosody, and voice quality measures.

**Results:**

Perceptual analyses revealed slow and variable speech rate, prolonged interword intervals and phonemes, imprecise consonants, distorted vowels, hypernasality, vocal tremor, harshness, breathiness, hoarseness, reduced loudness, and excess loudness variation in more than 50% of the participants. Acoustical analyses confirmed the presence of vocal tremor as well as numerically lower articulation rate, longer and more variable syllable duration, and lower smoothed cepstral peak prominence in more than 50% of participants compared to normative data from prior studies. Acoustical analyses classified one participant as ataxic and 14 participants as mixed hyperkinetic-ataxic.

**Discussion:**

This study revealed the presence of cerebellar signs of dysarthria in speakers with ET, primarily supporting a mixed hyperkinetic-ataxic dysarthria profile in ET. The data indicate that cerebellar dysfunction in ET does not spare speech and voice.

## Introduction

Essential tremor (ET) is a highly-prevalent neurological disease estimated to affect 7 million individuals in the United States [1]. Although the pathophysiology of ET is not fully understood, tremor likely arises from an abnormal cerebellar-thalamic-cortical pathway [2]. Two decades of postmortem studies have revealed degenerative pathology in the cerebellar cortex centered in and around the Purkinje cell population [3,4]. The pathophysiological involvement of the cerebellum in ET is mirrored by clinical features of the disease, which can involve a range of cerebellar signs [5], including intention tremor [5], eye motion abnormalities [6], ataxic gait [7], and problems with motor timing [8]. Yet it is largely unknown whether or how cerebellar dysfunction impacts speech in ET.

Dysarthria is not canonically regarded by neurologists as a feature of ET speech. On the other hand, the classic Darley Aronson, and Brown framework used by speech and language pathologists classifies the motor speech impairment in ET as hyperkinetic, grouping it with other disorders that involve involuntary movements [9]. In actuality, the features of dysarthria in ET have received minimal attention. To our knowledge, although several studies have investigated voice features in ET [10,11,12], only one study has investigated speech features specific to ET [13].

In three previous studies of voice in ET, participants were reported to have rhythmic modulation of fundamental frequency (*f_o_*) and intensity at a rate of 4–6 Hz in sustained vowels [11,12] consistent with vocal tremor, lower harmonics to noise ratio in vowels [10] that can be associated with a breathy voice quality [14], and lower intensity variability in sentences [10] that can be associated with reduced prosody [15]. In the single study that investigated speech patterns in ET (sample size = 25) [13], participants who had signs of cerebellar dysfunction exhibited increased syllable duration during diadochokinesis tasks (i.e., repeated syllable productions) associated with slow speech rate and commonly found in ataxic dysarthria [16], whereas speakers with ET who had only postural or simple kinetic tremor had typical syllable duration [13]. Although these findings suggest that speakers with ET may exhibit features of dysarthria consistent with hyperkinetic or ataxic dysarthria, the speakers’ ET diagnoses were uncertain given the limited diagnostic information presented in the studies [11,12] and the propensity for diagnostic misclassification in ET [17]. Moreover, there is considerable overlap in the features of hyperkinetic and ataxic dysarthria in the classic Darley, Aronson, & Brown framework [9,18], such that 9 of the 11 voice and speech features considered to be present or prominent in ataxic dysarthria overlap with the 30 voice and speech features that are present or prominent in hyperkinetic dysarthria summarized by Duffy (2020; Table 15–4) [19]. Thus, a more comprehensive analysis of dysarthria features across a broader range of speaking tasks is needed to characterize the features of dysarthria in speakers with confirmed ET.

Therefore, the aims of this study were to: 1) identify the perceptual features of dysarthria in ET, 2) verify the most prominent features of dysarthria using acoustical analyses of speech and voice, and 3) determine the percentage of participants who exhibit features of hyperkinetic dysarthria, ataxic dysarthria, or mixed hyperkinetic-ataxic dysarthria. We hypothesized that perceptual and acoustical analyses would reveal features of cerebellar dysfunction characteristic of ataxic dysarthria or mixed hyperkinetic-ataxic dysarthria. We also hypothesized that the perceptual features would be confirmed by acoustical analyses of speech. The findings of this study may clarify the perceptual and acoustical features of dysarthria specific to ET and to guide clinical assessment of dysarthria in ET. More fundamentally, they shed light on a biological question – does cerebellar dysfunction manifest clinically in ET speech and voice?

## Method

### Participants

Participants were recruited from two sources: [1] ET patients diagnosed by a movement disorders neurologist, who were attending an outpatient appointment in the Department of Neurology, University of Texas (UT) Southwestern Medical Center (recruitment from August to December 2022), and [2] individuals with ET enrolled in the Clinical-Pathological Study of Cognitive Impairment in Essential Tremor (COGNET), a nationwide longitudinal study of ET [20], who had a scheduled follow-up home visit (recruitment from January to June 2023). Eligible participants were over the age of 50 years and had been diagnosed with ET by a movement disorders neurologist. Exclusion criteria included a history of other movement disorders, stroke, chemotherapy treatment, laryngeal surgery, botulinum toxin injections to the larynx, or brain surgery for ET.

Twenty-seven participants were recruited and consented to participate in the study. Twelve participants whose audio recordings did not meet the criteria discussed below were excluded, resulting in a final sample of fifteen participants included in the final analyses. This study was approved by the UT Southwestern Institutional Review Board under two protocols: STU-2022-0108 and STU-2020-0564.

### Data Collection

#### Neurological Evaluation

All participants underwent a detailed tremor history and examination. Action tremor was rated by a senior movement disorders neurologist (E.D.L.) using the Washington Heights Inwood Genetic Study of ET rating scale, resulting in a total tremor score (range = 0–36) [21]. The ET diagnosis was confirmed by the same neurologist using Washington Heights Inwood Genetic Study of ET Criteria.

#### Speech Recordings

Audio recordings were collected in sound-treated offices in the Clinic Research Unit (CRU) or in quiet rooms in participants’ homes. To minimize background noise in participants’ homes, they were asked to turn off televisions, radios, or other devices; remove pets from the room; and request that family members refrain from speaking during the recording. Trained research staff asked participants to wear a head-mounted microphone (Shure WH20XLR) that was positioned 5 cm from the corner of the mouth and 45° off-axis. The microphone was connected to an audio-digital interface (Behringer U-PHORIA UM2) and routed to a laptop computer (Dell Latitude 3300 for recordings at CRU and Dell Precision 5530 for the at-home recordings) with Audacity (Audacity Team, December 2021, v 3.1.3) recording software. Participants received verbal and written instructions to perform a series of speaking tasks. They first sustained the vowel /ɑ/ with their softest voice for five seconds (s) across three trials with at least 5 s of silence between each. Next, they sustained the vowels /ɑ/ and /i/ with their comfortable pitch and loudness for 5 s across five trials. Participants then performed diadochokinesis tasks including repetition of /pʌ/, /tʌ/, /kʌ/, /pʌtʌkʌ/, /bʌ/, /dʌ/, /gʌ/, /bʌdʌgʌ/, and /ʔʌ/. Participants next read aloud five sentences that controlled for semantics and syntax from Kent et al. (2000) and five sentences with varied phonemic contexts from the Consensus Auditory-Perceptual Evaluation of Voice [22]. Finally, they spoke continuously for one minute about various topics (e.g., a favorite vacation or daily activities). All speech recordings were collected with a sampling rate of 44.1 kHz. The assessment duration was 20 minutes.

### Data Analysis

#### Audio Samples

Audio recordings were inspected in Praat (Boersma & Weenink; version 6.3.09) to assess signal quality, participants’ productions, and examiners’ instructions for each task in the speaking protocol. Audio recordings were excluded from analysis when participants did not produce at least four trials of each vowel, all ten sentences, and at least 30 s of continuous speech; when the signal amplitude was not sufficiently above the noise floor for auditory-perceptual analyses; and when examiners’ instructions deviated substantially from the protocol. Twelve participants’ recordings were excluded from analysis: two recorded in the CRU and 10 recorded in participant’ homes. Tasks in the included samples were manually segmented in Praat, and examiners’ speech was removed. All edited samples were then concatenated in Praat, RMS normalized in Audacity (Audacity Team, 2024, v.3.5.1) using default settings, and then extracted by participant and task in Praat.

#### Auditory-Perceptual Ratings

The last author, a licensed and certified speech-language pathologist with 8 years of clinical and 15 years of research experience in speech and voice, completed auditory-perceptual ratings for each participant’s speech samples. Audio samples were presented in a sound-treated room using a desktop computer (Dell, Inc., Precision 3630) with Praat and circumaural headphones (Sennheiser HD 280 Pro) with a Rolls HA43 headphone amplifier. The samples were presented at a comfortable level, which was maintained for all participants’ samples. The samples were presented in random order by participant but consistent order by task (vowels, sentences, spontaneous speaking). The rater played the samples as many times as needed to complete the ratings, which was no more than 5 times each.

As each sample was presented, the rater completed relevant sections of the Colorado Motor Speech Framework (CMSF) [23]. The CMSF is a tool for assessing motor speech disorders, which includes a checklist of perceptual features of speech and voice described by Duffy (2020) for differential diagnosis of dysarthria. Table 1 lists the perceptual features rated for different speaking tasks in each perceptual domain. The types of dysarthria associated with each feature were deleted from the CMSF form used for the ratings, and indications of the presence/absence of each feature in dysarthria were also obscured. After all ratings were completed, total counts of features were computed in the CMSF spreadsheet for each type of dysarthria (flaccid, unilateral upper motor neuron, spastic, hypokinetic, hyperkinetic, ataxic) and apraxia of speech.

**Table 1:** Perceptual Features Rated for Different Speaking Tasks in Each Perceptual Domain.


PERCEPTUAL DOMAIN	SPEAKING TASK	PERCEPTUAL FEATURES RATED

Speech timing	Sentences, spontaneous speech	Slow rate, fast rate, variable rate, short phrases, prolonged interword intervals, atypical pauses/silences, prolonged phonemes

Fluency	Sentences, spontaneous speech	Abnormal noises that interrupt speech or occur when patient is not speaking, stutter-like disfluencies, distorted substitutions or articulatory additions, syllable segregation

Prosody	Sentences, spontaneous speech	Reduced use of stress, prosodic excess or scanning, errors marking stress, monopitch, monoloudness

Articulation	Sentences, spontaneous speech	Imprecise consonants, distorted vowels, irregular articulatory breakdown, articulatory groping, telescoping, deterioration of speech during continuous speaking

Resonance	Sustained vowels, sentences, spontaneous speech	Audible nasal emission or nasal snorting, hyponasality, hypernasality

Voice Quality	Sustained vowels, sentences, spontaneous speech	Breathiness, aphonia, hoarseness, strained/strangled, diplophonia, harshness, voice stoppage, vocal tremor, rapid vocal flutter

Loudness	Sustained vowels, sentences, spontaneous speech	Reduced loudness, explosive loudness bursts, loudness decay, excess loudness variation


#### Acoustical Analyses

Audio files were manually edited in Praat to extract vowels and spontaneous speech for acoustical analysis. A multi-step automated acoustic analysis model separated staff’s and participant’s speech, transcribed the participant’s speech, and analyzed temporal and prosodic aspects of the participant’s speech. A speech diarization model [24] distinguished between the staff’s and participant’s speech and separated their speech, and a speech recognition (Wav2vec2) model [25] transcribed each participant’s speech. The speech transcription file was manually inspected for errors while visualizing the associated audio file in Praat. Any transcription errors (e.g., spelling errors, missing words, or missing transcription of filled pauses) were manually corrected. The Montreal Forced Aligner [26] was then used to align words in the corrected transcription file with words in the audio file and to generate separate word and phoneme tiers in a TextGrid file. The alignment of these tiers was inspected for errors, and the transcription was manually corrected as needed. Next, acoustical analyses of speech and voice were performed using a combination of Praat scripts and Python-based models. Acoustical analyses were selected that corresponded to the perceptual features commonly observed in the auditory-perceptual ratings of the participants’ spontaneous speech and vowel samples. These acoustical analyses of speech and voice features are described in Table 2. Signal-to-noise ratio (SNR) was calculated in Praat by measuring the difference in intensity between 1 s of the softest sustained vowel /ɑ/ produced at comfortable loudness and 1 s of the loudest background noise within the block of vowel productions.

**Table 2:** Acoustical Analyses of Speech and Voice Features.


PERCEPTUAL DOMAIN	PERCEPTUAL FEATURE	ACOUSTICAL CORRELATE	SPEAKING TASK	ANALYSIS TOOL	ANALYSIS DESCRIPTION

Speech timing	Slow rate of speech	Speech rate	Spontaneous speech	Publicly-available Praat script (Praat Vocal Toolkit) [54]	Number of syllables divided by the entire utterance duration (including silent and filled pauses and other dysfluencies like syllable repetitions) [34]

		Articulation rate	Spontaneous speech	Publicly-available Praat script (Praat Vocal Toolkit) [54]	The number of syllables divided by the phonation duration, which excluded silent pauses [34,54]

	Prolonged inter-word intervals	Silent pause duration	Spontaneous speech	Publicly-available Praat script (Praat Vocal Toolkit) [54]	Duration of silences at least 0.3 s long

		Filled pause duration	Spontaneous speech	Custom-written Python script	Number and duration of filled pauses (“uh”, “uhm”, etc.) obtained from transcribed .txt files and corresponding TextGrids generated using Montreal Forced Aligner

	Prolonged phonemes	Syllable duration	Spontaneous speech	Publicly-available Praat script (Praat Vocal Toolkit) [54]	Mean duration of syllables

	Variable rate of speech, imprecise consonants and other articulatory inaccuracies	Variability of syllable duration	Spontaneous speech	Custom-written Praat script	SD of syllable duration obtained from a TextGrid generated using Corretge (2021–2024).

Prosody	Prosodic excess	Variability of *f_o_*	Spontaneous speech	Custom-written Praat script.^1^	SD of *f_o_* computed using autocorrelation

Voice Quality	Vocal tremor	*f_o_* modulation rate and extent, intensity modulation rate and extent	Sustained vowels	Custom-written Praat scripts [55]	Number of cycles of *f_o_* modulation per second and magnitude of *f_o_* modulation for the middle 1 s segment of each sustained /ɑ/ and /i/^2^

	Harshness, Breathiness, Hoarseness	Smoothed cepstral peak prominence (CPPS)	Sustained vowels	Publicly-available Praat script (Praat Vocal Toolkit)	A measure of periodic energy in the voice signal from the middle 1 s segment of the second sustained /ɑ/ produced by each participant [56]


^1^ A template of this Praat script was generated using OpenAI (2025) using the prompt “generate a Praat script to calculate SD of fundamental frequency” and then modified by the first author. ^2^ Semi-automated analyses of vocal tremor were completed for participants who exhibited rhythmic modulation of *f_o_* or intensity based on visual inspection of the contours in Praat.

#### Statistical Analyses

Because this study did not include a control group, between-group statistical comparisons could not be completed. Instead, we computed descriptive statistics (means and standard deviations [SDs]) for all acoustical measures within our sample. To interpret these findings, we reviewed previously published studies that included healthy older controls and reported the mean, SD, or range of controls’ acoustical measures. While the control groups were not identical in age and sex to the present group of participants with ET, the reported ranges provided a reasonable comparison to normative data from older adults.

#### Data Availability Statement

Data that are not provided in the article because of space limitations may be anonymized and shared at the request of any qualified investigator for purposes of replicating procedures and results.

## Results

### Participants

Six male (mean age = 70.0 years; SD = 7.3; range = 62–82) and nine female participants with ET (mean age = 79.8 years; SD = 5.7; range = 68–88) were included. Six participants’ data were recorded in the CRU, and nine participants’ data were recorded in participants’ homes. The average age of tremor onset was 42.3 years (SD = 19.1, range = 16–62) for males and 43.7 years (SD = 23.6, range = 6–73) for females. The average duration of ET was 27.8 years (SD = 17.1, range = 3–36) for males and 36.1 years (SD = 24.8, range = 9–75) for females. Of the 15 participants, nine (60.0%) reported head tremor, and five (33.3%) reported voice tremor. The median total tremor score was 21 (mean = 20.6, standard deviation = 6.2), indicating tremor of moderate severity. Two participants had severe upper limb tremor (total tremor score > 30). All participants except two female and three male participants were on medication for ET. Of the 15 participants, five were taking propranolol, two were taking primidone, and three were taking both propranolol and primidone. Both of these medications are commonly used in the management of ET. Propranolol and primidone do not significantly affect speech or voice in most patients with ET [27,28,29]. Prolonged use of antipsychotic medications that block dopamine can cause tardive dyskinesia, which is associated with hyperkinetic dysarthria [19]. None of our participants were taking these medications, and none had tardive dyskinesia. Angiotensin-converting enzyme (ACE) inhibitors may cause coughing, which can affect voice quality [30,31,32]. Three participants were taking an ACE inhibitor (i.e., lisinopril, ramipril, enalapril).

### Auditory-Perceptual Ratings

**Figure 1:**
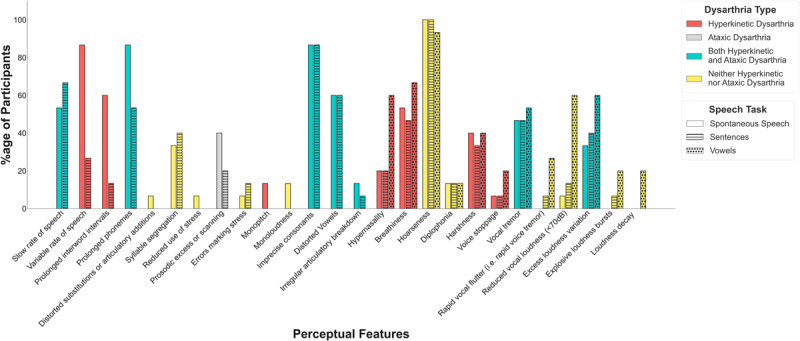
Perceptual Features of Dysarthria Across Speaking Tasks.

The percentages of participants exhibiting each perceptual feature of hyperkinetic dysarthria, ataxic dysarthria, or both hyperkinetic and ataxic dysarthria are shown in Figure 1. More than 50% of the participants exhibited slow rate of speech, variable rate of speech, prolonged interword intervals, prolonged phonemes, imprecise consonants, distorted vowels, hypernasality, vocal tremor, harshness, breathiness, hoarseness, reduced loudness, and excess loudness variation in at least one speaking task. Of these, the features that are considered present or prominent in hyperkinetic dysarthria, but not ataxic dysarthria included variable rate of speech (13 participants; 86.7%), prolonged interword intervals (9 participants; 60.0%), hypernasality (9 participants; 60.0%), harshness (8 participants; 53.3%), and breathiness (10 participants; 66.7%). The features that are considered present or prominent in ataxic dysarthria but not in hyperkinetic dysarthria included prosodic excess or scanning (6 participants; 40.0%). Telescoping of syllables, a feature of ataxic dysarthria, was not perceived in any participant. The features that are considered present or prominent in both hyperkinetic and ataxic dysarthria included slow rate of speech (10 participants; 66.7%), prolonged phonemes (13 participants; 86.7%), imprecise consonants (13 participants; 86.7%), distorted vowels (9 participants; 60.0%), vocal tremor (8 participants; 53.3%), and excess loudness variation (9 participants; 60.0%). The features that are not considered present or prominent in either hyperkinetic or ataxic dysarthria included hoarseness (15 participants; 100.0%) and reduced loudness (9 participants; 60.0%).

### Acoustical Analyses

#### Speech Timing

**Figure 2:**
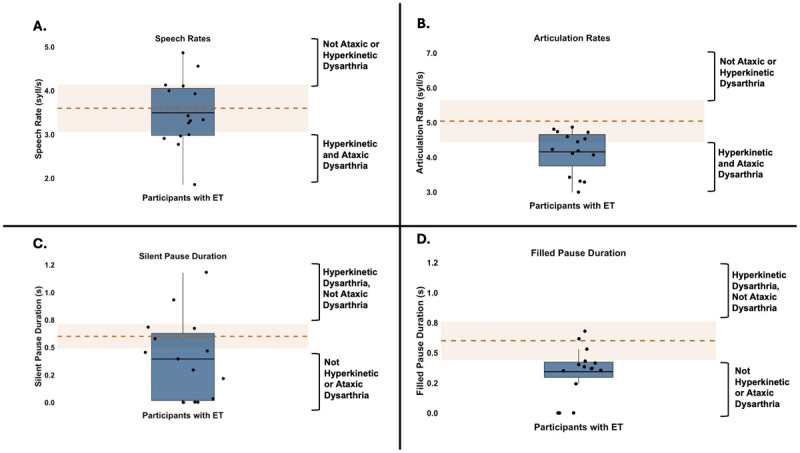
Speech Timing Measures in Spontaneous Speech Samples from Participants With ET. **A.** Speech rate (syllables/s). **B.** Articulation rate (syllables/s). **C.** Silent pause duration (s). **D.** Filled pause duration (s). The solid blue box represents the interquartile range of the speech timing measure, with the black dots representing individual participants and the black horizontal lines representing the group mean. The dashed orange line marks the normative mean, and the shaded orange region indicates the normative range (±1 SD from the mean). Square brackets on the y-axis indicate patterns associated with ataxic or hyperkinetic dysarthria.

The mean speech rate in the current sample was 3.5 syllables/s (*SD* = 0.78; range = 1.9–4.8). Healthy older adults aged 65–75 years had comparable speech rates (*M* = 3.6 syllables/s, *SD* = 0.5) [33]. Figure 2A shows the speech rates of the participants with ET as compared with the normative mean and SD from a previous study with neurotypical speakers [33]. Five (33.3%) participants exhibited speech rates numerically lower than the typical range, suggestive of both hyperkinetic and ataxic dysarthria.

The mean articulation rate in this sample was 4.1 syllables/s (*SD* = 0.6, range = 3.0–4.9). This aligns with articulation rates ranging from 3.6–5.0 syllables/s reported for spontaneous speech in healthy older adults aged 51- 65 years [34,35]. Figure 2B shows the articulation rates of the participants with ET as compared with the normative mean and SD from previous studies. Eight (53.3%) participants exhibited articulation rates numerically lower than the typical range, suggestive of both hyperkinetic and ataxic dysarthria.

Participants produced an average of 6 silent pauses (range = 0–12) and 3 filled pauses (range = 0–8) in each sample. The mean silent pause duration was 0.4 s (*SD* = 0.4, range = 0–1.2). Among the participants who exhibited silent pauses, the duration of silent pauses ranged from 0.3 s–3.0 s (S1). The mean filled pause duration was 0.3 s (*SD* = 0.2, range = 0–0.7). Healthy older adults aged 70–90 years had a numerically higher mean silent pause duration of 0.60 s (*SD* = 0.1) [36], and healthy older adults aged 58–81 years had numerically comparable filled pause duration of 0.3 s (*SD* = 0.2) [37]. Figures 2C and 2D show the mean silent and filled pause duration for the participants with ET as compared with the normative mean and SD from previous studies. Two participants exhibited silent pause durations numerically longer than the typical range, which is consistent with hyperkinetic dysarthria.

**Figure 3:**
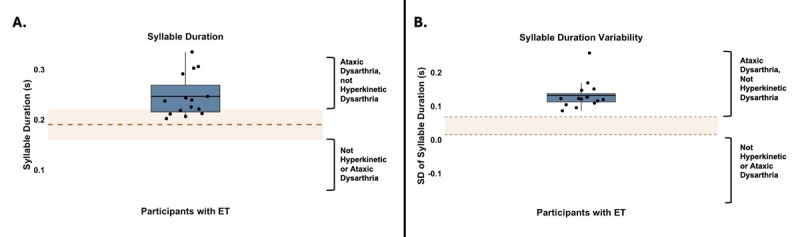
Syllable Duration Measures in Spontaneous Speech Samples from Participants with ET. **A.** Mean syllable duration **B.** SD of syllable duration The solid blue boxes represent the interquartile range, with the black dots representing individual participants and the black horizontal lines representing the group mean. The dashed orange line in A. marks the normative mean, and the shaded orange region indicates the normative range (±1 SD from the mean). Because the mean SD of syllable duration was not reported in previous studies, the shaded orange region indicates the normative range only in B. Square brackets on the y-axis indicate patterns associated with ataxic or hyperkinetic dysarthria.

The mean syllable duration of participants was 0.3 s (*SD* = 0.0, range = 0.2–0.3), and the mean SD of syllable duration of participants was 0.1 s (SD = 0.0, range = 0.1–0.3). Figure 3 shows the mean and SD of syllable duration for participants with ET presented alongside normative values from previous studies. Ten participants (66.7%) had mean syllable durations numerically longer than typical (M = 0.2 s, SD = 0.0) [38], and all 15 participants (100.0%) had SD of syllable duration numerically greater than the reported range for older healthy adults (0.02–0.07 s) [39]. Both patterns are consistent with ataxic dysarthria.

#### Prosody

**Figure 4:**
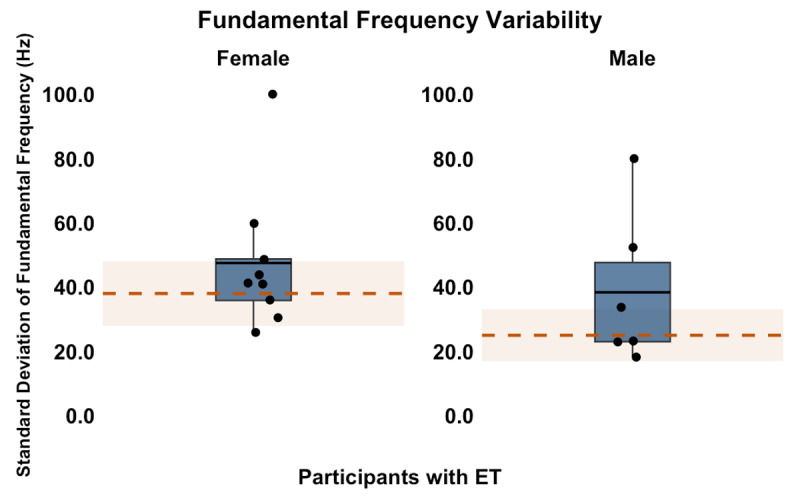
The *f_o_* Variability in Spontaneous Speech Samples of Female and Male Participants with ET. The solid blue boxes represent the interquartile ranges, with the black dots representing individual participants and the black horizontal lines representing the group mean. The dashed orange line marks the normative mean, and the shaded orange region indicates the normative range (±1 SD from the mean). Square brackets on the y-axis indicate ranges associated with ataxic or hyperkinetic dysarthria.

The average *f_o_* variability of female and male participants was 47.5 Hz (*SD* = 22.0, range = 26.2–99.9) and 38.4 Hz (*SD* = 23.7, range = 18.2–79.9), respectively. Because normative values for *f_o_* variability differed by sex, results were stratified accordingly. Reported means for healthy older female adults were 38.0 Hz (SD = 10), and for healthy older male adults were 25.0 Hz (SD = 8) [40]. As shown in Figure 4A, three female and three male (40.0%) participants had *f_o_* variability numerically higher than the normative range, which is suggestive of ataxic dysarthria.

#### Voice Quality

Visual analyses of modulation in *f_o_* and intensity contours revealed that 8 (53.3%) of participants had vocal tremor, which is a feature of both hyperkinetic and ataxic dysarthria. Based on semi-automated acoustical analyses of the modulation patterns, the average *f_o_* and intensity modulation rates were 4.6 and 5.2 Hz, respectively. The average *f_o_* and intensity modulation extents were 24.1 and 57.4%, respectively. We contextualized these findings with published normative data from speakers with Huntington’s disease (typically associated with hyperkinetic dysarthria) and cerebellar disease (typically associated with ataxic dysarthria) as described in the Supplementary Material. S2 shows the rates and extents of *f_o_* and intensity modulation in the current sample, which did not consistently align with hyperkinetic or ataxic dysarthria.

**Figure 5:**
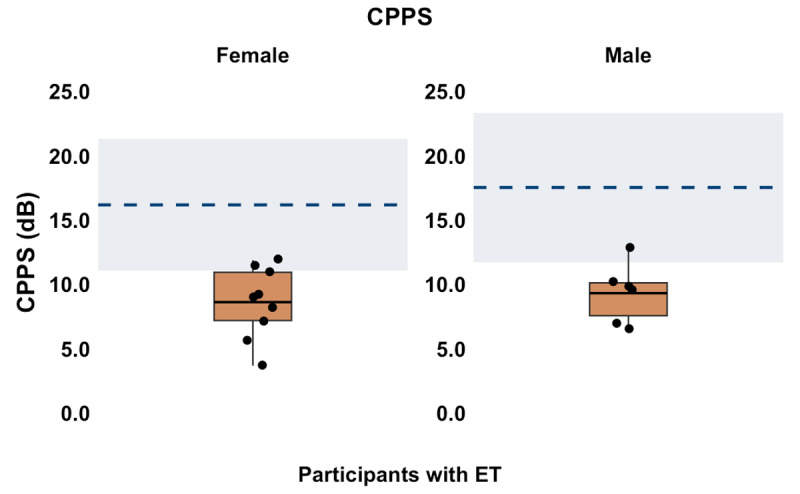
CPPS for Female and Male Participants from Sustained Vowels. The solid orange boxes represent the interquartile range, with the black dots representing individual participants and the black horizontal lines representing the group mean. The dashed blue line marks the normative mean, and the shaded blue region indicates the normative range (±2 SD from the mean).

Lastly, the mean CPPS value from sustained vowel productions was 8.6 dB (SD = 2.7, range = 3.7–11.9) for female participants, and 9.3 dB (SD = 2.3, range = 6.6–12.8) for male participants. Of the 15 participants, 12 (80.0%) participants including seven female and five male participants had CPPS values below the normative values reported for adult females (M = 16.2 dB, SD = 2.6) and adult males (M = 17.5 dB, SD = 2.9) [41], respectively. The reduced CPPS values could be due to harshness [42] or transient breathiness [43], which are characteristics of hyperkinetic dysarthria. Figure 5 shows the CPPS values obtained in the current participant sample.

#### SNR

SNR values ranged from 10.9 dB to 35.9 dB, with a mean of 21.5 dB.

#### Classification of Dysarthria Type Based on Acoustical Analyses

To illustrate the acoustical patterns within and across participants, Table 3 presents participant-level means of the acoustical features illustrated in Figures 2, 3, 4, 5. In addition, S4 presents a heatmap showing z-scores and normalized values of acoustical analyses for each participant. A classification logic (S3) was implemented in R (v4.4.0; R Core Team, 2024) and described in the Supplementary Materials. Based on the classification criteria, one participant was classified as having ataxic dysarthria, and fourteen participants were classified as having mixed hyperkinetic-ataxic, which is represented in the final column of S4.

**Table 3:** Participant-level means of the acoustical features illustrated in Figures 2, 3, 4, 5. Dysarthria feature classifications are indicated in parentheses: (H) hyperkinetic, (A) ataxic, and (B) both hyperkinetic and ataxic.


PARTICIPANT	SEX	AGE (years)	SPEECH RATE (syllables/s)	ARTICULATION RATE (syllables/s)	AVG SILENT PAUSE DURATION (s)	AVG FILLED PAUSE DURATION (s)	AVG SYLLABLE DURATION (s)	SD OF SYLLABLE DURATION	SD OF *f_o_*	CPPS (dB)

P1	F	68	3.0	3.4 (B)	0.4	0.2	0.3 (A)	0.1 (A)	35.8	10.9

P2	M	62	4.9	4.9	0.0	0.4	0.2	0.1 (A)	79.9 (A)	12.8

P3	M	65	3.9	4.5	0.5	0.4	0.2 (A)	0.1 (A)	18.2	9.6 (H)

P4	F	77	4.0	4.7	0.6	0.4	0.2	0.1 (A)	26.2	11.6

P5	M	80	3.3	3.3 (B)	0.0	0.5	0.3 (A)	0.3 (A)	44.1	9.3 (H)

P6	M	69	4.1	4.1 (B)	0.0	0.6	0.2 (A)	0.1 (A)	22.9	10.2 (H)

P7	M	82	2.8 (B)	4.6	0.9 (H)	0.0	0.2	0.1 (A)	52.3 (A)	9.8 (H)

P8	F	82	3.3	4.2 (B)	0.7	0.7	0.2 (A)	0.1 (A)	48.7 (A)	7.2 (H)

P9	F	76	3.0	3.0 (B)	0.0	0.4	0.3 (A)	0.2 (A)	40.8	3.7 (H)

P10	F	83	4.1	4.8	0.7	0.0	0.2	0.1 (A)	60.0 (A)	9.1 (H)

P11	F	81	1.9 (B)	3.3 (B)	1.2 (H)	0.4	0.3 (A)	0.2 (A)	30.2	11.9

P12	F	83	2.9 (B)	4.2 (B)	0.5	0.0	0.2 (A)	0.1 (A)	41.5	5.7 (H)

P13	F	88	4.6	4.7	0.0	0.4	0.2	0.1 (A)	99.9 (A)	8.2 (H)

P14	M	67	3.4	4.5	0.3	0.4	0.2 (A)	0.1 (A)	23.2	6.5 (H)

P15	M	75	3.3	4.1 (B)	0.2	0.4	0.2 (A)	0.1 (A)	33.6 (A)	6.9 (H)


## Discussion

The features of dysarthria in ET have been largely understudied. The present study aimed to: 1) identify the perceptual features of dysarthria in ET, 2) verify the most prominent features of dysarthria using acoustical analyses of speech and voice, and 3) determine the percentage of participants who exhibit features of hyperkinetic dysarthria, ataxic dysarthria, or mixed hyperkinetic-ataxic dysarthria. We hypothesized that perceptual and acoustical analyses of speech and voice in participants with ET would reveal cerebellar signs consistent with ataxic dysarthria or mixed hyperkinetic-ataxic dysarthria.

Fifteen participants with ET were included in the study and performed speech tasks while audio recordings were collected. Perceptual analyses revealed slow rate of speech, variable rate of speech, prolonged interword intervals, prolonged phonemes, imprecise consonants, distorted vowels, hypernasality, vocal tremor, harshness, breathiness, hoarseness, reduced loudness, and excess loudness variation in more than 50.0% of the participants. Acoustical analyses confirmed the presence of vocal tremor as well as numerically lower articulation rate, longer mean syllable duration, higher SD of syllable duration, and lower CPPS in more than 50% of participants compared to normative data from prior studies. Based on the classification logic implemented in this study using the acoustical analyses, one participant was classified as ataxic and the remaining 14 participants were classified as mixed hyperkinetic-ataxic. To further explore the patterns of dysarthria in ET, we examined the association between the perceptual and acoustical features in the participant sample.

The perception of slow speech rate, which is a feature of both hyperkinetic and ataxic dysarthria, may be linked acoustically to reduced speech rate or articulation rate, increased syllable duration [44], or prolonged silent or filled pauses [45]. However, the acoustical analyses in the current study revealed that most participants exhibited numerically faster than typical speech rates and shorter than typical pauses, suggesting these features were not likely to have contributed to the perception of slow speech rate. Instead, the numerically lower articulation rates or increased syllable duration may have been the primary acoustical correlates underlying the perceptual judgment of slow speech rate [46,47]. While reduced articulation rate is consistent with both hyperkinetic and ataxic dysarthria [19], increased syllable duration aligns with the perception of prolonged phonemes, which is an established characteristic of ataxic dysarthria. This feature may reflect underlying hypotonia, which slows muscle activation and speech movements [48]. Alternatively, cerebellar dysfunction may lead to delayed motor responses to auditory feedback, resulting in extended segment durations [49].

It is worthwhile to note that some participants exhibited a wide range of silent pause duration, with approximately 46.7% producing individual pauses that were numerically longer than the normative mean (S1), and 13.3% producing average silent pause durations that were numerically longer than the normative mean. For these participants, prolonged silent pauses may have contributed to the perception of slow speech rate. Silent pause duration may also serve as an acoustical correlate of prolonged interword intervals, which is a prominent feature of hyperkinetic dysarthria. This disruption is thought to result from involuntary movement interruptions or impaired control over speech initiation and timing [9,18]. Together, these findings suggest that participants with markedly prolonged silent pauses may have a hyperkinetic dysarthria profile. In contrast, the numerically higher SD of syllable duration in our sample is characteristic of ataxic dysarthria [16] reflecting difficulties in speech motor control. Previous studies have shown that ataxic dysarthria is characterized by temporal and spatial inaccuracy in segmental production, leading to articulatory inaccuracies and irregular syllable timing [16], suggesting that some participants with ET in our study may have ataxic dysarthria. Thus, the speech timing patterns in the current sample of participants may support either a mixed hyperkinetic-ataxic or an ataxic dysarthria profile.

Another common observation in ataxic dysarthria is prosodic excess or increased pitch variability. In the current sample of participants, this was reflected in the numerically increased SD of *f_o_*. This may be due to reduced coordination between the laryngeal and respiratory systems [16,19] or impaired laryngeal sensory feedback, which can produce irregular vocal fold vibration and unstable pitch [50]. Thus, the prosody findings provide further evidence that some of the participants with ET in the current sample may have ataxic dysarthria.

To determine if increased pitch variability was related to vocal tremor, we conducted analyses of *f_o_* and intensity modulation, which revealed that participants exhibited a 3.8–5.6 Hz rate of *f_o_* modulation. While three of these participants also showed numerically increased *f_o_* variability, five others with vocal tremor did not. Additionally, there are no established thresholds in tremor measures that reliably differentiate between hyperkinetic and ataxic dysarthria [51,52]. However, the reduced CPPS of the current sample of participants aligns with the perceptual findings of breathiness [43], a transient characteristic of hyperkinetic dysarthria [23]. Thus, these voice quality findings could support a hyperkinetic or ataxic dysarthria profile in the speakers with ET.

These speech and voice patterns reveal features of cerebellar dysfunction and primarily support a mixed hyperkinetic-ataxic dysarthria profile in ET. Prominent perceptual features of hyperkinetic dysarthria were noted in a large percentage of participants, such as prolonged interword intervals and intermittent breathiness. The prominent perceptual feature of ataxic dysarthria prosodic excess or scanning was also noted. The patterns in acoustical analyses classified 14 out of 15 participants as having mixed hyperkinetic-ataxic dysarthria. One participant exhibited only ataxic features, suggesting that a subgroup of speakers with ET may have a pure ataxic profile.

### Limitations and Future Directions

The current study investigated the perceptual and acoustical patterns of dysarthria in ET, revealing primarily mixed hyperkinetic-ataxic patterns in ET and motivating future research. A limitation of this study was that the auditory-perceptual ratings were completed by one expert listener, the last author who had knowledge of the study questions. However, the ratings only served to guide the selection of objective acoustical analyses, and acoustical analyses patterns confirmed several perceptual patterns. Future studies should include additional raters and analyze formant frequencies for vowel distortions, intensity for prosody and loudness, and speech timing patterns in diadochokinesis tasks.

A second limitation of this study was the presence of background noise in the recordings and lower than optimal SNR. However, acoustical analyses that are sensitive to noise like CPPS can be reliably measured at 20 dB SNR, and depending on the noise type, even at 10 dB SNR [53]. Additionally, all participants’ recordings were higher than 10 dB SNR, and nearly half had SNR higher than 20 dB. Despite this, future studies should reduce background noise to improve acoustical analyses. Also, regarding the acoustical analyses, although we attempted to align our acoustic methods with prior studies, analysis procedures and demographic mismatches (e.g., age, sex, gender) may have influenced results. For example, pause duration was measured for typical speakers during a story retell task [36], which has a higher cognitive load than conversational speaking and may elicit longer pauses. Thus, this task difference could have masked differences between typical speakers and speakers with ET. Future studies should include age-, sex-, and gender-matched control groups and use identical speech tasks and acoustic analyses to ensure more accurate comparisons.

Lastly, while the classic Darley, Aronson, & Brown framework was used to interpret and characterize the perceptual and acoustical findings in this study, there were differences between the classic framework and the CMSF checklist used for perceptual ratings. For example, variable rate of speech and monopitch are considered to be ‘present’ in hyperkinetic dysarthria but not ataxic dysarthria in the Darley, Aronson, & Brown framework; whereas, these features are considered in the CMSF to be ‘present’ in both hyperkinetic and ataxic dysarthria. Thus, further research is needed to identify which features should be considered as ‘present but not distinguishing’ in hyperkinetic and ataxic dysarthria.

## Conclusion

Clinical and postmortem literature implicates cerebellar involvement in ET. Although cerebellar signs in ET are well established for limb and eye movements, their impact on speech has not been established. Dysarthria is not canonically regarded by neurologists as a feature of ET speech. However, the motor speech impairment in ET is classified as hyperkinetic dysarthria in the classic Darley, Aronson, & Brown framework. In the present study, perceptual and acoustical analyses revealed predominantly mixed hyperkinetic–ataxic features of speech and voice in participants with ET, aligning with the corpus of existing evidence supporting cerebellar involvement in ET. The data indicate that cerebellar dysfunction in ET does not spare speech and voice.

## Additional File

The additional file for this article can be found as follows:

10.5334/tohm.1180.s1 Supplemental Materials. Additional acoustical analyses and classifications of dysarthria types.
