# Supplementary material for: Perceptual and Acoustical Features of Dysarthria in Essential Tremor: An Observational Study that Expands the Cerebellar Features of Essential Tremor
Source: Tremor Other Hyperkinet Mov (N Y). 2026 May 6;16:30. doi: 10.5334/tohm.1180 (PMC13155085; doi:10.5334/tohm.1180)
Supplement: Supplemental Materials. — Additional acoustical analyses and classifications of dysarthria types. [file tohm-16-1-1180-s1.pdf]

Supplementary Material

To explore features associated with hyperkinetic dysarthria in the current sample of participants, we examined silent pause durations for each participant. Boxplots for individual participants are presented in Figure S1.

Figure S1

Boxplots of silent pause duration for each participant with ET. The shaded orange band indicates the normative range (±1 SD) and the dashed line marks the normative mean. Participants with silent pause durations above the normative range have longer pausing patterns associated with hyperkinetic dysarthria.

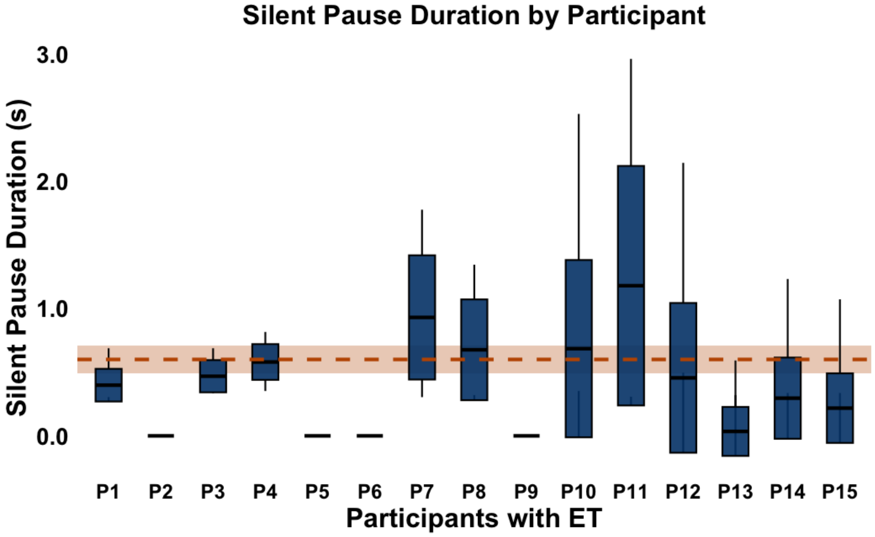

All participant's f_o modulation rates were within the range reported for speakers with Huntington's disease (M = 4.5, SD = 3.2) (1) and cerebellar disease (M = 3.7, SD = 2.1) (2). Three participants' intensity modulation rates were within the range reported for speakers with Huntington's disease (M = 3.2, SD = 1.5) (1), and all participants had numerically higher rates of intensity modulation than in speakers with cerebellar disease (M = 2.6, SD = 0.8) (2). Two participants were within the reported f_o modulation extent for speakers with Huntington's disease (M = 1.2, SD = 0.8) (1), and one participant was within the reported f_o modulation extent for speakers with cerebellar disease (M = 1.0, SD = 0.4) (2). All participant's intensity modulation extents numerically exceeded the reported range (M = 5.7, SD = 2.9) for speakers with Huntington's disease (1), and only one participant was within the reported range for speakers with cerebellar disease (M = 8.7, SD = 4.7). Figure S2 shows the rates and extents of f_o and intensity modulation in the current sample.

Figure S2. Vocal Tremor Measures in Sustained Vowels Produced by Participants With ET.

A. f_o modulation rate
B. f_o modulation extent
C. intensity modulation rate
D. intensity modulation extent

The solid orange boxes represent the interquartile range, with the black dots representing individual participants. The dashed blue line and the dashed orange line mark the normative means for hyperkinetic and ataxic dysarthria, respectively, and the shaded regions indicate the normative range (±1 SD from the mean).

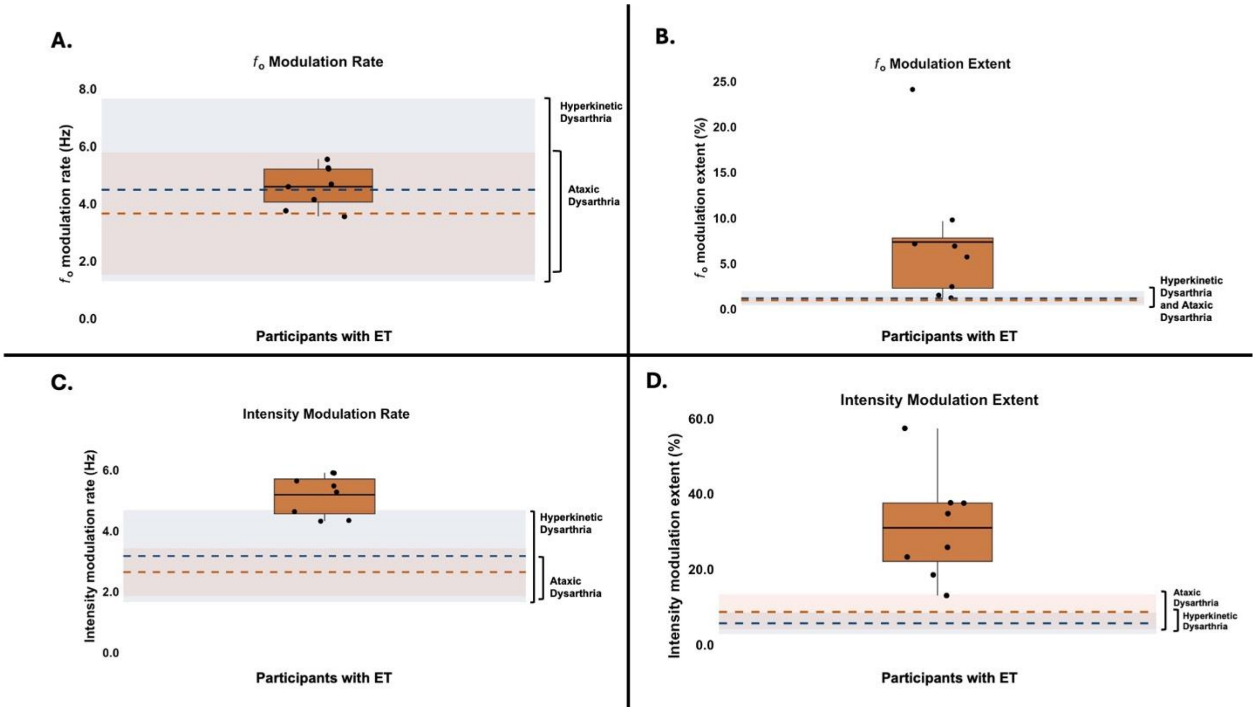

Figure S3 represents the decision logic implemented for classifying speakers with hyperkinetic, ataxic, or mixed hyperkinetic-ataxic dysarthria. Speakers were classified as based on whether their z-scores deviated by ±1 SD from the normative mean across selected acoustical features. Speakers with numerically increased pause durations, typical syllable duration, typical f_o variability, and reduced CPPS were classified as hyperkinetic; speakers with typical pause durations, high f_o variability, increased syllable duration, increased variability in syllable duration were classified as ataxic; and speakers meeting criteria for both patterns were classified as mixed hyperkinetic-ataxic.

Figure S3. Classification Logic to Classify Participants into Hyperkinetic, Ataxic or Mixed Based on Acoustic Features.

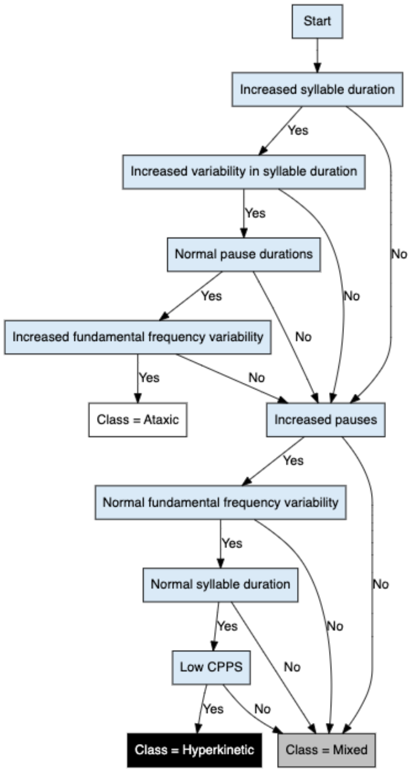

Z-scores were calculated for each feature using the mean and SD reported in previous studies. Positive z-scores reflect above-average values, while negative z-scores reflect below-average values. The SD of syllable duration values were normalized based on the available normative range, with values mapped from 0 (minimum) to 1 (maximum), to enable visualization in the absence of a reported mean and SD. The heatmap in Figure S4 also has two additional columns to indicate each speaker's average f_o and intensity modulation extent in percentage.

Figure S4. Heatmap Displaying Acoustical Analyses for Each Speaker.

The column for SD of syllable duration represents scaled values normalized to a 0–1 range based on the normative range. All other columns represent z-scored acoustic features. Orange shades represent values that are higher than the normative average, while blue shades represent values that are lower than the normative average. Two additional columns show each speaker's average f_o and intensity modulation extents, with higher rates represented by darker shades of orange. The rightmost column indicates the dysarthria classification.

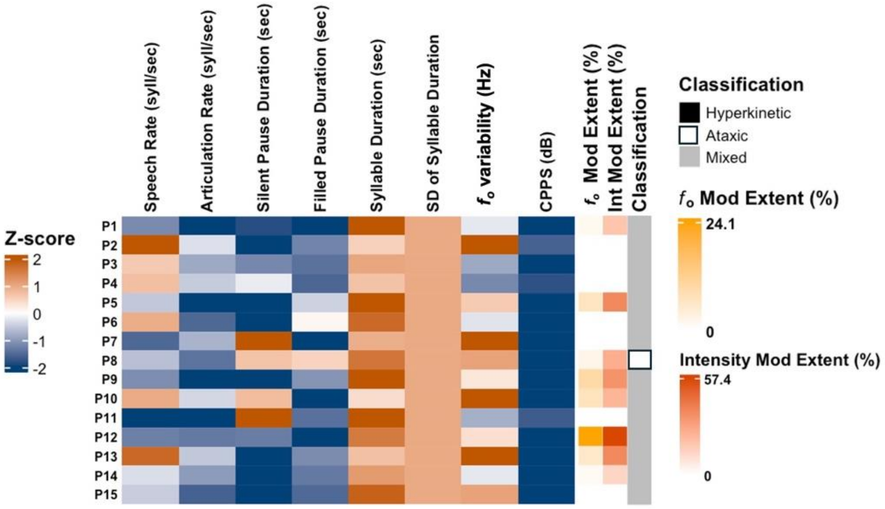

References

1. Velasco García MJ, Cobeta I, Martín G, Alonso-Navarro H, Jimenez-Jimenez FJ. Acoustic Analysis of Voice in Huntington’s Disease Patients. Journal of Voice. 2011 Mar;25(2):208–17. doi:10.1016/j.jvoice.2009.08.007

2. Boutsen F, Duffy JR, Dimassi H, Christman SS. Long-Term Phonatory Instability in Ataxic Dysarthria. Folia Phoniatr Logop. 2011;63(4):216–20. doi:10.1159/000319971
